# Phenotypic and Genotypic Characterization of Pan-Drug-Resistant *Klebsiella pneumoniae* Isolated in Qatar

**DOI:** 10.3390/antibiotics13030275

**Published:** 2024-03-19

**Authors:** Mazen A. Sid Ahmed, Jemal M. Hamid, Ahmed M. M. Hassan, Sulieman Abu Jarir, Emad Bashir Ibrahim, Hamad Abdel Hadi

**Affiliations:** 1 Laboratory Services, Department of Public Health, Philadelphia, PA 19146, USA; mazen.sidahmed@phila.gov; 2 Division of Microbiology, Department of Pathology and Laboratory Medicine, Hamad Medical Corporation, Doha P.O. Box 3050, Qatar; jhamid@hamad.qa (J.M.H.); ahassan46@hamad.qa (A.M.M.H.); eelmagboul@hamad.qa (E.B.I.); 3 Division of Infectious Diseases, Communicable Diseases Centre, Hamad Medical Corporation, Doha P.O. Box 3050, Qatar; sabujarir@hamad.qa; 4 Biomedical Research Centre, Qatar University, Doha P.O. Box 2713, Qatar

**Keywords:** antimicrobial resistance, AMR, Gram-negative bacteria (GNB), *Klebsiella pneumoniae*, NDM-5

## Abstract

In secondary healthcare, carbapenem-resistant Enterobacterales (CREs), such as those observed in *Klebsiella pneumoniae*, are a global public health priority with significant clinical outcomes. In this study, we described the clinical, phenotypic, and genotypic characteristics of three pan-drug-resistant (PDR) isolates that demonstrated extended resistance to conventional and novel antimicrobials. All patients had risk factors for the acquisition of multidrug-resistant organisms, while microbiological susceptibility testing showed resistance to all conventional antimicrobials. Advanced susceptibility testing demonstrated resistance to broad agents, such as ceftazidime-avibactam, ceftolozane–tazobactam, and meropenem–vaborbactam. Nevertheless, all isolates were susceptible to cefiderocol, suggested as one of the novel antimicrobials that demonstrated potent in vitro activity against resistant Gram-negative bacteria, including CREs, pointing toward its potential therapeutic role for PDR pathogens. Expanded genomic studies revealed multiple antimicrobial-resistant genes (ARGs), including *bla*_NMD-5_ and *bla*_OXA_ derivative types, as well as a mutated outer membrane porin protein (OmpK37).

## 1. Introduction

Carbapenem-resistant Enterobacterales (CREs) are resistant Gram-negative bacteria, which are recognized as a public health priority with significant clinical and economic outcomes [1,2]. Importantly, *Klebsiella pneumonia* has been associated with a wide spectrum of community- and hospital-acquired infections (HAIs), including urinary, intra-abdominal, and respiratory tract infections, with significant morbidity and mortality [3,4]. The pathogen frequently affects vulnerable patients, such as those with chronic comorbidities, the immunocompromised, or those with critical diseases, particularly following invasive surgery or procedures at critical care units [3,5]. Regionally and globally, recent decades have witnessed alarming challenges related to *K. pneumoniae* due to its recognized capabilities as a prime community and nosocomial infection, as well as its ability to accumulate an extensive array of antibiotic resistance genes (ARGs), with special attention directed toward hypervirulent clones that manifest as multidrug-resistant (MDR) and extensively drug-resistant (XDR) variants, leading to ominous outcomes [6].

To evade antimicrobials, isolates of *K. pneumoniae* exhibit various complex resistance mechanisms that are acquired through random genetic mutations or horizontally acquired through mobile genetic elements, which can be subsequently amplified through the upregulation of resistance genes or continuously disseminated [7]. The generated mutations not only lead to antimicrobial resistance but also maximize the pathogen virulence through conferring resistance to phagocytosis, increasing capsular attachments and cellular invasion, resulting in enhanced ability for dissemination and increased mortality [8]. Worryingly, recent reports highlighted the emergence of extensive and pan-drug-resistant (PDR) strains of *K. pneumoniae* from different countries, limiting further treatment options [9,10,11,12]. Previous genomic studies of *K. pneumoniae* in Qatar established the clonality of ST383, ST196, and ST146 in healthcare settings, where they predominately carried the carbapenem-resistant genes (CRGs) *bla*_NDM_ and *bla*_OXA 48_ [13]. Furthermore, studies conducted in this field describe a large group of ARGs that impede the therapeutic function of broad-spectrum antimicrobials, such as carbapenems, as well as novel β-lactam–β-lactamase inhibitors (BLBLIs), which are considered the ultimate management agents for serious infections secondary to Gram-negative bacteria (GNB) [14,15].

Due to regional variations, in the Gulf countries, there is a lack of data linking pathogens’ phenotypic and genotypic characteristics to obtain a reliable molecular epidemiology of AMR in the region. In the current focused study, we outlined clinical, phenotypic, and genotypic characteristics of three PDR *K. pneumoniae* isolates, aiming to describe the underlying resistance mechanisms using observations obtained from antimicrobial susceptibility tests (ASTs), as well as whole-genome sequencing (WGS).

## 2. Results

### 2.1. Clinical Characteristics

All three cases were hospital-acquired and isolated from patients who were admitted to critical care units; aged above 50; and had multiple risk factors for MDR infections, including recent systemic antimicrobial therapy (three out of three), hospitalization or outpatient hospital attendance within the previous 90 days (two out of three), invasive medical devices (two out of three), and history of MDR infection or colonization within the 90 days prior to hospitalization (two out of three), as outlined in Supplementary Table S1. The most frequent underlying comorbidities were diabetes mellitus (two out of three) and immune suppression following a lung transplant (one out of three). Meropenem, tigecycline, and colistin were used to treat the patient with the KP1 isolate with new signs of infection, while the two colonized patients (isolates KP2 and KP3) did not receive specific therapy, since evaluated as colonization.

### 2.2. Microbiological Characteristic

The ASTs for the three isolates (KP1, KP2, and KP3) are outlined in Table 1; they demonstrated resistance to all conventional anti-GNB agents from different antimicrobial classes. Additional ASTs for novel antimicrobials revealed susceptibility to cefiderocol (three out of three), while two out of three isolates were susceptible to eravacycline, omadacycline, and ceftazidime/avibactam, and none to plazomicin, ceftolozane/tazobactam, imipenem/relebactam, and meropenem/vaborbactam.

### 2.3. Genomic Characteristics

The genomic size of the three PDR *K. pneumoniae* isolates KP1, KP2, and KP3 were 5,617,763 base pairs (bp), 5,617,835 bp, and 5,677,828 bp, respectively. Two of the three PDR *K. pneumoniae* isolates belonged to sequence type ST231, while the last one belonged to ST383. The genomic data of the three PDR *K. pneumoniae* isolates revealed that they possessed seven different β-lactamase genes from all classes; class A ESBL (CTX-M-15 and TEM-1) in all isolates (three out of three), while class A β-lactamase (CTX-M-14 and SHV-1), class B β-lactamase (NDM-5), and class D β-lactamase (OXA-232) were present in two out of three (Table 2). Different enzyme-encoding genes highlighting antibiotic target alteration, such as mutant *gyrA*, penicillin-binding protein (PBP3), and 16S rRNA methyltransferase (rmtF) (G1405), and *K. pneumoniae* mutated outer membrane porin with reduced permeability (*OmpK37*) were detected in all isolates, in addition to active efflux pump complexes, such as emrB and baeR, as depicted in Table 3 and Table 4.

## 3. Discussion

In order to understand the underlying mechanisms that lead to antimicrobial resistance (AMR), detailed evaluations of extensive-drug-resistant (XDR) and pan-drug-resistant (PDR) organisms are of paramount importance [17]. The problem of AMR is more evident in Gram-negative bacteria (GNB) because of the accumulated and diverse resistance mechanism that peaks in carbapenem resistance (CR) pathogens [18]. The subset of CREs represented by *K. pneumoniae* is one of the leading challenges implicated in healthcare-associated infections (HAIs), with significant ominous clinical and economic outcomes [3]. Resistance in *K. pneumoniae* is increasingly reported, particularly in patients with chronic comorbidities, immune-suppressions, previous antimicrobial exposure, and prolonged hospital or critical care length of stay [19]. The described cases were from patients with all risk factors for AMR and HAIs, namely, prolonged hospital stay; prior antimicrobial therapy; invasive devices; and chronic comorbidities, including immune suppression, as highlighted in S1 [20]. In addition to the outlined risk factors, it must be highlighted that all of the isolated cases were from patients admitted to critical care units where multidrug-resistant organisms peak in healthcare settings. The most vulnerable are elderly patients, as in our reported cases, with multiple comorbidities who undergo invasive procedures, such as central lines and intubation, and are usually treated with broad-spectrum antimicrobials, which propagates continuous resistance that flourishes in the surrounding environment, constituting an additional risk factor that is not easily controlled through the established concepts of infection control and prevention [21].

The outlined microbiological and genomic studies of the reported cases of PDR *K. pneumoniae* demonstrated extensive resistance to conventional antimicrobials, such as all third- and fourth-generation cephalosporins, monobactams, carbapenems, aminoglycosides, and quinolones, as well as polymyxins, represented by colistin (Table 1). Upon genomic characterization, two of the three isolates belonged to the notorious epidemic clones: *K. pneumoniae* ST231, which was first described in Southeast Asia and has been associated with healthcare outbreaks, as well as demonstrated diverse ARGs spanning major classes, particularly *bla*_OXA-48_ [22]. In a previous surveillance study from Qatar of 149 CREs, 8.6% of isolates were of the same epidemic clone, explaining its endemicity; which was affirmed in a recently updated and detailed study [13,23]. Additionally, class A extended-spectrum β-lactamases (ESBLs) were represented by *bla*_CXM-14_ and *bla*_CXM-15_, *bla*_SHV-1_, and *bla*_TEM-1_. Globally, *bla*_CXM-15_ is the most widely distributed ARG in GNB, including reports from the Gulf countries [24,25,26]. Furthermore, the worrying carbapenemase *bla*_NDM-5_ of class B metallo-beta-lactamase (MBL) was present in the KP1 isolate. This notorious ARG is an offspring of *bla*_NDM_, which was first described in 2012 and is carried horizontally through mobile genetic elements (MGEs); widely known as being capable of producing carbapenamases that can inactivate all basic and advanced β-lactamases, including cephalosporins and carbapenems but remain susceptible to monobactams, which is one of the few therapeutic agents that are capable of overcoming MBLs, though it is critically vulnerable to other ESBLs. The notorious *bla*_NDM-1_ ARG is endemic in the region, where it is one of the most prevalent genes amongst CREs [27,28] Nevertheless, *bla*_NDM-5_ has been reported mainly in CR *E. coli* from Greece, Japan, China, and Latin America, and is usually associated with highly virulent strains [29,30,31]. From a large collection of CR *E. coli*, the increasingly reported *bla*_NDM-5_ has been linked to plasmid-mediated resistance genes, which can be assumed to be horizontally acquired by MDR strains of *K. pneumoniae* [32]. Reported studies demonstrated the IncX3-type *bla*_NDM-5_ carrying a plasmid that can easily transfer resistance to susceptible strains [33]. The ARGs were also isolated from companion animals, which emphasizes the concept of one health approach to integrate the impact of interactions between humans, animals, and the environment [34]. In the Gulf region, the commonest reported carbapenem-resistance genes are *bla*_OXA48_ and *bla*_NDM_, with a historic preponderance of the former [27,28]. With the gradual introduction of novel antimicrobials, namely, β-lactam–β-lactamase inhibitor (BLBLI) agents, in the region such as ceftazidime-avibactam and ceftolozane-tazobactam, which have activity against *bla*_OXA_ type CREs but remain vulnerable to MBLs coupled with the absence of effective therapeutic agents for MBLs, this might result in shifting regional epidemiology, which will need further supporting surveillance in the coming years [35].

While examining the phenotypic patterns, characteristically, the first (KP1) showed utmost resistance to novel combinations, including β-lactam–β-lactamase inhibitors (BLBLIs), such as ceftazidime–avibactam, ceftolozane–tazobactam, and meropenem–vaborbactam, while the other two were susceptible to ceftazidime–avibactam but resistant to ceftolozane–tazobactam. Avibactam is a potent BLBLI that inhibits classes A, C, and D, including OXA types, but is incapable of inactivating enzymes encoded by class B β-lactamases, such *bla*_NDM_ and *bla*_VIM_, as in KP1, while ceftolozane is an advanced cephalosporin that overcomes many enzymes encoded by ARGs but remains susceptible to broad-spectrum ones, including OXA-types ARGs [36]. The fact that the two other isolates KP2 and KP3 lacked *bla*_NDM_ but harbored *bla*_OXA 232_ demonstrated broad BLBLI discordance (KP2 and KP3 were sensitive to ceftazidime-avibactam but both were resistant to ceftolozane–tazobactam), indicating that the ARG *bla*_OXA 232_ can be suppressed by avibactam but not tazobactam, as previously observed [37]. Similarly, both meropenem/vaborbactam and imipenem/relebactam were inferior, supporting the observation that the novel agent does not cover *bla*_NDM_ and *bla*_OXA_ type isolates, as previously described [38].The recommended management of CREs harboring class B MBLs, such as *bla*_NDM_, is usually complex and involves combination therapy with agents that are capable of overcoming the genotypic resistance, such as aztreonam–avibactam or cefiderocol, with or without the addition of additional antimicrobials, such as polymyxins, tigecycline, or eravacycline [39].

Distinctively, two of our PDR isolates were sensitive to the drugs that are promising alternative options. It is also intriguing to notice that all PDR isolates were susceptible to cefiderocol, which is one of the promising novel antimicrobials that demonstrated potent in vitro activity against XDR and PDR Gram-negative strains spanning all β-lactamase classes but needs supporting data toward clinical efficacy [40,41]. Cefiderocol is a novel synthetic siderophore antimicrobial that hijacks bacterial iron-transporting mechanisms to traverse the microbial cell wall and eventually leads to cell lysis through interference with cell wall synthesis. Its distinct ability to resist cell-wall-based β-lactamases, including classes A, B, C, and D, in addition to its unique ability to overcome bacterial efflux pumps and porin channels, make it a promising Trojan horse that is capable of overcoming different mechanisms of bacterial AMR. This antimicrobial is of significant potential interest since it has remarkable in vitro antimicrobial activity, particularly for notorious organisms, such as MDR *Pseudomonas aeruginosa* and carbapenem-resistant *Acinetobacter baumannii*, as well as *Stenotrophomonas maltophila.* Nevertheless, translation of the ASTs needs supporting clinical data since, in some observational studies, cefiderocol was linked to increased mortality, such as the one observed with carbapenem-resistant *Acinetobacter baumannii* [42,43]. Similarly, both eravacycline and omadacycline showed activity against two of the three PDR strains (KP2 and KP3 in Table 2). These advanced and novel derivative agents of the tetracycline group are of focused interest, particularly for the coverage of resistant strains from intrabdominal infections, though they are limited for serious invasive diseases, such as bloodstream infections based on observed pharmaco-kinetic (PK) and pharmacodynamic (PD) characteristics [41,44,45]. As highlighted, one of the reported cases was treated with high-dose tigecycline, which is currently one of the limited options to treat CREs, including PDR. Since there were many limitations for the PK and PD for tigecycline, the newer same-class eravacyline demonstrated multiple promising in vitro and some clinical results against CREs, including MBLs with promising results [46]. Currently, the novel drug is mainly licensed to treat complicated intrabdominal infections and awaits additional clinical results, particularly for complicated hospital-acquired pneumonia [47].

While polymyxin resistance is extremely rare in CREs (less than 5% in most regions), intriguingly, the three isolates showed colistin resistance [15]. Polymyxins are not natural targets for ARGs since they act primarily and independently at the cell wall levels of GNB by destroying structural lipopolysaccharides. The reported resistance is mainly driven by the plasmid-mediated mobile colistin resistance (*mcr*-resistant gene) first described in China in 2015 in *E. coli* and then described in most Enterobacterales [48]. The fact that no *mcr*-related genes were identified in the isolates points toward other outer-membrane resistance mechanisms, which merit further pursuit. Previous studies of colistin-resistant *K. pneumoniae* lacking the *mcr* resistance gene explored the role of mutations in the two-component membrane system of the *phoPQ* and *pmrAB* [49]. Additionally, all isolates harbored Rifampin ADP-ribosyl transferase (*Arr*), which confers rifamycin resistance when used as an adjuvant therapy, though this was not tested in the study. Furthermore, all the isolates harbored OXA-type carbapenemases, such as *bla*_OXA 48_, which is closely related to the resistance gene *bla*_OXA 232_, which has five-point mutations from *bla*_OXA 48_ and both are similarly capable of inactivating carbapenems [50]. It is worth pointing out that the OXA type, together with class B NDM carbapenemases, are the most frequent carbapenemases in the Middle East and Gulf regions [23,25].

Characteristically, genomic characterization revealed mutations at the outer membrane porin (OMP) permeability channels with the expressions of *K. pneumoniae*-associated OmpK35 mutation that usually lead to a decrease in permeability for several hydrophilic antimicrobials, such as β-lactams, including carbapenems; fluoroquinolone; tetracycline and its derivatives glycylcycline, represented by tigecycline; as well as rifamycin. Furthermore, it has been reported that mutated bacteria expressing OmpK35 is associated with increased virulence. Additionally, previous studies demonstrated that alternation of the OMP cellular mutations leads to the restoration of lost antimicrobial activities [51].

In addition to the common ARGs, genotypic characterization revealed the occurrence of other mechanisms, such as aminoglycosides modifying enzymes (AMEs), macrolide phosphotransferase (MPT), and fosfomycin thiol transferase resistance genes, that might be linked to reported phenotypic patterns. For PDR GNB, the co-occurrence and transmission of multiple resistance mechanisms is a well-recognized phenomenon that frequently leads to the propagation of AMR [52].

Although this study tried to elucidate the underlying mechanism of resistance while focusing on *Klebsiella pneumoniae*, it is restricted by some limitations that should be taken into consideration for the validity of scientific evaluation. It must be highlighted that two of the *Klebsiella pneumoniae* isolates were evaluated as colonizers rather than a true infection, which limits its clinical evaluation, particularly regarding options for therapeutic interventions. Additionally, the genomic study focused on ARGs rather than combining them with virulence factors, which was not possible to explore because of the small collection and paucity of invasive diseases. Nevertheless, the relationship between bacterial AMR and virulence was found to be elusive, even for comprehensive studies. Despite these limitations, this study expanded our knowledge for the fascinating study of the mechanisms of resistance, particularly for GNB, which should pave the way to overcome its challenges.

## 4. Material and Methods

### 4.1. Definitions

A hospital-acquired infection (HAI) is defined as an infection acquired following 48 h of hospital admission, while colonization is defined as isolation of an organism from non-sterile sites and is not evaluated as an infection requiring no antimicrobial therapy.

### 4.2. Identification and Susceptibility Testing

Phenotypic characterizations were performed using a BD Phoenix^TM^ automated system (BD diagnostics, Durham, NC, USA), while bacterial identification and confirmation were performed using matrix-assisted laser desorption ionization time of flight mass spectrometry (MALDI-TOF MS) of Bruker Daltonics MALDI Biotyper (Billerica, MA, USA) according to the manufacturer’s recommendations. The standards for the identification of ESBL include resistance to third-generation cephalosporins, such as ceftriaxone or ceftazidime, with MIC > 2 µg/mL confirmed through double disc diffusion methods that include inhibition by a co-amoxiclav disc, as widely described in laboratory guidelines [53]. Additional ASTs for fosfomycin, cefiderocol, plazomicin, omadacycline, eravacycline, doxycycline, meropenem/vaborbactam, ceftazidime/avibactamimipenem/relebactam, and ceftolozane/tazobactam were performed using MIC Test Strips (Liofilchem^®^, Diagnostics, Roseto degli Abruzzi, Italy), while broth microdilution was used for the colistin susceptibility testing (ComASP Colistin, Liofilchem, Roseto degli Abruzzi, Italy). *Escherichia coli* ATCC 25922, *E. coli* ATCC 35218, and *Pseudomonas aeruginosa* ATCC 27853 were used as the controls. Susceptibility reporting was based on the recommendations of the CLSI at the time [53] PDR *K. pneumoniae* isolates were defined as having in vitro non-susceptibility to all routinely and conventionally tested anti-Gram-negative antimicrobial agents, excluding additional advanced susceptibility tests [54]. WGS was performed by Eurofins GATC Biotech GmbH, Konstanz, Germany, using the Illumina HiSeq 2000 system (Illumina, San Diego, CA, USA). Annotations were performed using the PATRIC RASTtk-enabled Genome Annotation Service [55]. ARGs were predicted using the Comprehensive Antibiotic Resistance Database (CARD) version 1.2.0 (McMaster University, Hamilton, ON, Canada) [56].

### 4.3. Ethical Approval

The study was approved by the Medical Research Centre (MRC) and Research and Ethics Committee (protocol: MRC-04-22-522) at Hamad Medical Corporation, Doha, Qatar, which abides by the local and international standards of ethics in medical research, including patient consent, data anonymity and management.

## 5. Conclusions

The clinical, phenotypic, and genotypic characterization of three PDR *K. pneumoniae* revealed multiple risk factors for the acquisition of healthcare-associated MDR pathogens, resulting in extensive resistance profiles, while examining the genomic studies revealed multiple underlying ARGs associated with phenotypic patterns. For PDR *K. pnuemoniae*, novel antimicrobial agents, such as cefiderocol, omadacycline, and eravacycline, are potential therapeutic agents that need further clinical evaluation.

## Figures and Tables

**Table 1:** Antimicrobial susceptibility profile of three pan-drug-resistant *K. pneumonia* isolates against conventional and novel antimicrobials.

Antimicrobial Class	Antimicrobial Drug	Isolate Number					
		KP1		KP2		KP3	
**Tested by BD Phoenix**							
Penicillins	Ampicillin	>16	R	>16	R	>16	R
Cephalosporins	Cefazolin	−	R	−	R	−	R
	Cefepime	>16	R	>16	R	>16	R
	Cefoxitin	>16	R	>16	R	>16	R
	Ceftazidime	>16	R	>16	R	>16	R
	Ceftriaxone	>32	R	>32	R	>32	R
	Cefuroxime	>16	R	>16	R	>16	R
	Cephalothin	>16	R	>16	R	>16	R
Monobactam	Aztreonam	>16	R	>16	R	>16	R
Carbapenems	Ertapenem	>4	R	>4	R	>4	R
	Imipenem	>8	R	>8	R	>8	R
	Meropenem	>8	R	>8	R	>8	R
β-lactam–β-lactamase inhibitors	Amoxicillin/clavulanate	>16/8	R	>16/8	R	>16/8	R
	Piperacillin/tazobactam	>64/4	R	>64/4	R	>64/4	R
Aminoglycosides	Amikacin	>32	R	>32	R	>32	R
	Gentamicin	>8	R	>8	R	>8	R
Fluoroquinolones	Ciprofloxacin	>2	R	>2	R	>2	R
	Levofloxacin	>4	R	>4	R	>4	R
	Nitrofurantoin	>64	R	>64	R	>64	R
Folate pathway inhibitors	Trimethoprim/sulfamethoxazole	>4/76	R	>4/76	R	>4/76	R
Glycylcyclines	** Tigecycline	4	R	2	I	2	I
**Additional Tested Antimicrobials Using MIC Test Strip**							
Fosfomycin	*** Fosfomycin	48	R	48	R	256	R
Cephalosporins	Cefiderocol	0.38	S	0.38	S	0.094	S
Aminoglycosides	Plazomicin	256	R	256	R	256	R
Tetracycline	Omadacycline	32	R	3	S	3	S
	Eravacycline	32	R	0.75	S	1.5	S
	Doxycycline	32	R	2	S	32	R
New β-lactam–β-lactamase inhibitors	Ceftazidime/avibactam	256	R	0.75	S	1	S
	Imipenem/relebactam	32	R	2	I	2	I
	Ceftolozane/tazobactam	256	R	256	R	16	R
	Meropenem/vaborbactam	32	R	12	I	8	I
**Tested Using Broth Microdilution Method**							
Polymyxin	Colistin *	16	R	16	R	8	R

Novel antimicrobial agents included cefiderocol, plazomycin, omadacyline, eravacycline, ceftazidime–avibactam, ceftolozane–tazobactam imipenem/relebactam, and meropenem/vaborbactam. * Colistin: antimicrobial susceptibility tested through broth microdilution methods. ** There are no CLSI break points for tigecycline, and thus, FDA-approved ones were used: Resistance (R) > 8, susceptible (S) < 2, and in-between are intermediate (I) [16]. *** There is no agreed consensus on fosfomycin breakpoints for GNB; the results were interpreted according to local experience.

**Table 2:** Sequence types and enzymatic genotypic profiles of PDR *K. pneumoniae* isolates.

Isolate Number (Sequence Type)		KP1 (ST383)	KP2 (ST231)	KP3 (ST231)
Resistance genes	Gene Family	Gene Presence (% Identity)		
AAC(6′)-Ib	AAC(3), AAC(6′)	Yes (100)	Yes (100)	Yes (100)
aadA	Amimonglycoside 3″-nucleotidyltransferases; ANT(3″)	VIM, Deletion b of E231 (99.23)	−	−
aadA2	ANT(3″)	−	Yes (100)	Yes (100)
APH(3′)-Ia	Aminoglycoside 3′-phosphotransferases; APH(3′)	L19M, R27K, N48D, A77E (98.52)	−	−
APH (3″)-Ib	APH(3″)	L116S (99.63)	−	−
APH (3′)-VI	APH (3′)	Yes (100)	−	−
APH (6)-Id	APH (6)	Q259E (99.64)	−	−
CTX-M-14	Class A β-lactamase	Yes (100)	−	−
CTX-M-15	Class A β-lactamase	Yes (100)	Yes (100)	Yes (100)
SHV-1	Class A β-lactamase	Yes (100)	−	Yes (100)
TEM-1	Class A β-lactamase	Yes (100)	Yes (100)	Yes (100)
NDM-5	Class B β-lactamase	Yes (100)	−	−
OXA-232	Class D β-lactamase	−	Yes (100)	Yes (100)
OXA-48	Class D β-lactamase	Yes (100)	−	−
arr-2	Rifampin ADP-ribosyl transferase (Arr)	Yes (100)	Yes (100)	Yes (100)
BRP (MBL)	Bleomycin resistant protein	−	−	−
catI	Acetyltransferase (CAT)	−	Yes (100)	Yes (100)
FosA6	Fosfomycin thiol transferase	Q130P, Q139E (98.56)	A86V, I91V, Q130P (97.84)	A86V, I91V, Q130P (97.84)
mphA	Macrolide phosphotransferase (MPH)	Yes (100)	Yes (100)	Yes (100)
mphE	Macrolide phosphotransferase (MPH)	Yes (100)	−	−
**Disc diffusion methods**	ESBL	Detected	Detected	Detected

Disk-diffusion-based screening methods for extended-spectrum β-lactamases.

**Table 3:** Sequence types and genotypic profiles of encoding enzymes of PDR *Klebsiella pneumoniae* isolates.

Isolate Number (Sequence Type)		KP1 (ST383)	KP2 (ST231)	KP3 (ST231)
Resistance Gene	Drug Class	Gene Presence (% Identity)		
**Antibiotic Target Alteration**				
16S rRNA methyltransferase (armA), (G1405)	Aminoglycoside	Yes (92.74)	−	−
Erm 23S ribosomal RNA methyltransferase (ErmB)	Lincosamide, macrolide, streptogramin	Yes (97.96)	Yes (97.96)	Yes (97.96)
EF-Tu mutants	Pulvomycin	Yes (97.97)	Yes (98.06)	Yes (98.06)
gyrA	Nybomycin, fluoroquinolone	Yes (95.67)	Yes (95.67)	Yes (92.23)
marR mutant	Cephalosporin, fluoroquinolone, penam, phenicol, glycylcycline, tetracycline, rifamycin, triclosan	Yes (84.03)	Yes (84.03)	Yes (84.03)
parC	Fluoroquinolone	Yes (94.41)	Yes (94.41)	Yes (94.41)
UhpT with mutation	Fosfomycin	Yes (95.03)	Yes (95.25)	Yes (95.25)
PBP3	β-lactam	Yes (52.37)	Yes (52.37)	Yes (52.37)
16S rRNA methyltransferase (rmtF), (G1405)	Aminoglycoside	Yes (98.36)	Yes (100)	Yes (100)
**Antibiotic Target Protection**				
ABC-F ATP-binding cassette ribosomal protection protein (msrE)	Macrolide antibiotic, streptogramin	Yes (100)	−	−
QqnrS2	Fluoroquinolone	Yes (100)	−	Yes (100)
ABC-F ATP-binding cassette ribosomal protection protein (vgaC)	Streptogramin, pleuromutilin	Yes (100)	Yes (91.89)	Yes (91.78, 83.78) *
**Antibiotic Target Replacement**				
Trimethoprim-resistant dihydrofolate reductase (dfr); dfrA12	Diaminopyrimidine	Yes (100)	Yes (100)	Yes (100)
dfrA5	Diaminopyrimidine	Yes (100)	−	−
Sulfonamide resistant (sul1)	Sulfonamide, sulfone	Yes (100)	Yes (100)	Yes (100)
Sulfonamide resistant (sul2)	Sulfonamide, sulfone	Yes (100)	−	−
**Reduced Permeability to Antibiotic**				
*Klebsiella pneumoniae* porin with reduced permeability (OmpK37)	β-lactams	Yes (99.47)	Yes (94.01)	Yes (94.01)
General bacterial porin with reduced permeability (marA)	β-lactam, fluoroquinolone, glycylcycline, triclosan, phenicol, tetracycline, rifamycin	Yes (92.74)	Yes (92.74)	Yes (92.74)

* Two different vgaC genes were detected.

**Table 4:** Occurrence of genotypic profile of efflux pump complexes and their regulators for *Klebsiella pneumoniae*.

Isolate Number (Sequence Type)			KP1 (ST383)	KP2 (ST231)	KP3 (ST231)
	Gene Family	Drug Class	Present or Absent		
**Efflux Pump Complexes**					
msbA	ABC ^a^	Nitroimidazole	+	+	+
emrB	MFS ^b^	Fluoroquinolone	+	+	+
QepA4	MFS	Quinolone and fluoroquinolone antibiotics	+	+	−
tet(A)	MFS	Tetracycline, glycylcycline	+	+	−
tet(C)	MFS	Tetracycline	+	−	−
tetR	MFS	Tetracycline	+	+	−
adeF	RND ^c^	Fluoroquinolone, tetracycline	+	+	+
baeR	RND	Aminoglycoside	+	+	+
oqxA	RND	Fluoroquinolone, nitrofuran, tetracycline, glycylcycline	+	+	+
**Efflux Pump Regulators**					
CRP	RND	Macrolide, fluoroquinolone, penam	+	+	+
emrR	MFS	Fluoroquinolone	+	+	+
H-NS	MFS, RND	Cephamycin, cephalosporin, fluoroquinolone, tetracycline, penam	+	+	+

^a^ ATP-binding cassette (ABC) antibiotic efflux pump. ^b^ Major facilitator superfamily (MFS) antibiotic efflux pump. ^c^ Resistance-nodulation-cell division (RND) antibiotic efflux pump.

## Data Availability

We highlight that the strictest confidence was maintained for data collection, as well as access during the study. Data were not shared at any level with any individuals not authorized to access the research material. Data can be made available upon a reasonable request to the authors following permission from the Medical Research Center at HMC. We fully understand that the use of confidential data for personal purposes is prohibited.

## Supplementary Materials

The following supporting information can be downloaded at: https://www.mdpi.com/article/10.3390/antibiotics13030275/s1, Table S1: Demographic, clinical characteristics and outcomes of three patients colonized or infected with PDR *K. pneumoniae.*
