# Supplementary material for: Phenotypic and Genotypic Characterization of Pan-Drug-Resistant Klebsiella pneumoniae Isolated in Qatar
Source: Antibiotics (Basel). 2024 Mar 19;13(3):275. doi: 10.3390/antibiotics13030275 (PMC10967633; doi:10.3390/antibiotics13030275)
Supplement: Supplementary file 1 [file antibiotics-13-00275-s001.zip › antibiotics-2853065-supplementary.pdf]

Supplementary Materials

Table S1. Demographic, clinical characteristics and outcomes of three patients colonized or infected with PDR *K. pneumoniae.*

Characteristics	KP1 (ST383)	KP2 (ST231)	KP3 (ST231)
Age	66	51	72
Gender	Male	Female	Male
Location	Critical Care Unit	Critical Care Unit	Critical Care Unit
Isolation site	Urine tract	Respiratory tract *	Respiratory tract *
Common associated underlying conditions			
Extensive health care contact [a]	No	Yes	Yes
History of antibiotic exposure within 90 days prior to hospital admission	Yes	Yes	Yes
Invasive devices [b]	Yes	Yes	No
Diabetes mellitus	Yes	Yes	No
History of MDR infection or colonization within prior 90 days	No	Yes	Yes
Co-infection with other microorganisms [c]	Yes	Yes	No
Heart failure	Yes	No	No
Chronic lung disease	No	No	Yes
Post-transplantation	No	Yes	Yes
Chronic liver disease	No	Yes	No
Acquisition	Hospital	Hospital	Hospital
Disease evaluation	Colonization	Colonization	Sepsis

Antibiotic treatment			
Meropenem [d]	No	No	Yes
Tigecycline [e]	No	No	Yes
Colistin nebulizer	No	No	Yes

Patients and pathogens identification: KP refer to patients 1,2 and 3 while ST refer to pathogens sequence types. [a] Extensive health care contact involves regular visits to outpatient medical facilities, a regular home visit by home care teams, hospitalization within the preceding 90 days, or residency in a long-term care facility. [b] Invasive devices involve central line, Foley's catheter, and tracheostomy. [c] Co-infection is associated with the following organisms: other strains of MDR *K. pneumonia* and MDR *Acinetobacter baumannii*. [d] High dose meropenem 2000 mg/mL IV. [e] High dose tigecycline 200 mg loading followed by 100 mg IV every 12 hours. * Respiratory tract samples were tracheal aspirates
